# Analysis of the key enzymes of butyric and acetic acid fermentation in biogas reactors

**DOI:** 10.1111/1751-7915.12299

**Published:** 2015-06-18

**Authors:** Christina Gabris, Frank R Bengelsdorf, Peter Dürre

**Affiliations:** Institute of Microbiology and Biotechnology, University of UlmAlbert-Einstein-Allee 11, D-89081, Ulm, Germany

## Abstract

This study aimed at the investigation of the mechanisms of acidogenesis, which is a key process during anaerobic digestion. To expose possible bottlenecks, specific activities of the key enzymes of acidification, such as acetate kinase (Ack, 0.23–0.99 U mg^−1^ protein), butyrate kinase (Buk, < 0.03 U mg^−1^ protein) and butyryl-CoA:acetate-CoA transferase (But, 3.24–7.64 U mg^−1^ protein), were determined in cell free extracts of biogas reactor content from three different biogas reactors. Furthermore, the detection of Ack was successful via Western blot analysis. Quantification of corresponding functional genes encoding Buk (*buk*) and But (*but*) was not feasible, although an amplification was possible. Thus, phylogenetic trees were constructed based on respective gene fragments. Four new clades of possible butyrate-producing bacteria were postulated, as well as bacteria of the genera *Roseburia* or *Clostridium* identified. The low Buk activity was in contrast to the high specific But activity in the analysed samples. Butyrate formation via Buk activity does barely occur in the investigated biogas reactor. Specific enzyme activities (Ack, Buk and But) in samples drawn from three different biogas reactors correlated with ammonia and ammonium concentrations (NH_3_ and NH_4_^+^-N), and a negative dependency can be postulated. Thus, high concentrations of NH_3_ and NH_4_^+^-N may lead to a bottleneck in acidogenesis due to decreased specific acidogenic enzyme activities.

## Introduction

The biochemical process of methane formation in biogas reactors can be divided into four phases: (i) hydrolysis of complex polymers, (ii) acidogenesis, (iii) acetogenesis and (iv) methanation (Weiland, 2010). In the first and second steps, the main products sugars and amino acids are formed, as well as volatile fatty acids (VFA) such as acetate, butyrate and propionate. These VFAs play a key role in biogas reactors. If the hydrolysation of organic material occurs too fast, VFA are accumulating, which has an effect on the growth of acetoclastic and hydrogenotrophic methanogens and their ability to produce methane (McCarty and McKinney, 1961; Kroeker *et al*., 1979; Chen *et al*., 1980; Weiland, 2010). The most widespread acetate-producing pathway comprises the enzymes phosphotransacetylase (Pta) and acetate kinase (Ack), which convert acetyl-CoA to acetate (Ljungdahl, 1986). This pathway can be found in many different prokaryotes, e.g. *Roseburia sp*. (Duncan *et al*., 2002), *Clostridium acetobutylicum* (Winzer *et al*., 1997) or *Methanosarcina thermophila* (Gorrell and Ferry, 2007). Butyrate-producing bacteria can be found, e.g., in marine sediments (Sørensen *et al*., 1981), and the human colon (Pryde *et al*., 2002; Louis *et al*., 2004; 2010), e.g. species related to *Eubacterium*, *Roseburia* or *Faecalibacterium*. These bacteria use one of the four major bacterial butyrate synthesis pathways – acetyl-CoA, glutarate, lysine and 4-aminobutyrate pathway – which all have in common crotonyl-CoA as an intermediate (Vital *et al*., 2014). By activity of butyryl-CoA dehydrogenase electron-transferring flavoprotein complex, butyryl-CoA is formed from crotonyl-CoA. Finally, butyryl-CoA is transformed to butyrate by a number of butyryl-CoA transferases, e.g. butyryl-CoA:acetate-CoA transferase (But), which uses acetate as co-substrate (Duncan *et al*., 2002). Butyryl-CoA can also be phosphorylated and transformed to butyrate by butyrate kinase (Buk) with simultaneous production of ATP (Diez-Gonzalez *et al*., 1999).

Here, we studied the specific activity of the enzymes Ack, Buk and But in three different biogas reactors. Specific activities can be used to determine the amount of the respective catalyst present in reactor content under different process conditions (Table 1). Furthermore, the presence and the quantity of the conserved genes *buk* and *but* were analysed in different samples of one biogas reactor.

**Table 1:** Samples and operating conditions of the analysed biogas reactors

Date month/year	Sample	pH	VFA^a^^b^ (g HAceq l^−1^)	TAC^c^ (g CaCO_3_ l^−1^)	VFA/TAC ratio	NH_4_^+^-N (g l^−1^)	DS^d^ (g kg^−1^)	oDS^e^ (g kg^−1^)
BR^f^ 1								
03/13	A	7.9	3.28	18.7	0.18	3.14	103.0	78.3
07/13	B	8.0	3.99	21.7	0.18	4.38	104.0	78.4
08/13	C	8.2	4.47	21.2	0.21	4.45	93.1	68.3
01/14	D	8.1	5.36	25.7	0.21	4.90	109.0	79.6
04/14	E	8.0	3.47	19.0	0.18	3.37	88.0	66.7
10/14	F	8.0	3.75	19.0	0.20	3.20	98.7	71.5
01/15	G	7.9	3.37	17.9	0.19	3.06	96.6	68.8
BR 2								
11/14	A1	8.1	4.61	22.8	0.20	4.28	117.0	87.9
01/15	B1	8.1	4.24	23.9	0.18	4.54	118.0	87.4
BR 3								
11/14	A2	7.5	2.17	8.4	0.26	0.92	83.5	65.3
02/15	B2	7.5	2.48	10.3	0.24	1.49	85.4	65.9

a Volatile fatty acids.

b Acetate 0.13–1.11 g liter^−1^, butyrate and propionate < 0.19 g liter^−1^.

c Total anorganic carbonate buffer.

d Dry solids.

e Organic dry solids.

f Biogas reactor.

## Results

Specific enzyme activities of the key enzymes Ack, Buk and But were measured photometrically by using cell free extracts of biogas reactor content obtained by ultrasonic pulsation (Table 2, Table S1). Specific activity of Ack was on average 0.41 Units (U) mg^−1^ protein in cell free extracts of samples A-G of the biogas reactor content of BR 1. Furthermore, only low specific Buk activity (< 0.02 U mg^−1^ protein) was measurable in the same samples. In contrast, high specific But activity (on average, 5.92 U mg^−1^ protein) was determined in samples B-G of BR 1. In cell free extracts of biogas reactor content obtained from BR 2 and BR 3 (samples A1, B1, A2 and B2) showed the same tendency of specific enzymatic activities. Specific Buk activity was lowest (< 0.03 U mg^−1^ protein), followed by specific Ack activity (0.23 and 0.99 mU mg^−1^ protein) and specific But activity (3.24 and 7.64 U mg^−1^ protein) respectively. Statistical analysis showed a correlation between specific enzyme activities and total ammonia and ammonium concentrations in reactor content of three different biogas reactors (Kendall’s rank coefficient τ = −1).

**Table 2:** Specific enzyme activities in U mg^−1^ protein of Ack, Buk and But in cell free extracts of biogas reactor content, *C. glutamicum* ATCC 13032 13AK and *C. kluyveri* DSM 555

Specific enzyme activities (U mg^−1^ protein)				NH4^+^-N (g l^−1^)/NH3 (g l^−1^)^g^
Sample	Ack	Buk	But	
BR 1	0.41 ± 0.10^a^	< 0.02^a^	5.92 ± 1.59^b^	3.79/0.51
BR 2	0.23^c^	< 0.01^c^	3.24^c^	4.41/0.71
BR 3	0.99^d^	< 0.03^d^	7.64^d^	1.21/0.05
Control	< 0.001^e^	< 0.001^e^	< 0.001^f^	

Results of BR 1 show the mean values and standard deviations from at least five biological replicates with four technical replicates. Mean values of ammonia concentrations for NH4+-N and NH3 in g l^−1^.

a Samples A–G.

b Samples B–G.

c Samples A1, B1.

d Samples A2, B2.

e *C. glutamicum* ATCC 13032 13AK.

f *C. kluyveri* DSM 555.

g 3 calculated (Angelidaki and Ahring, 1993).

**Figure 1:**
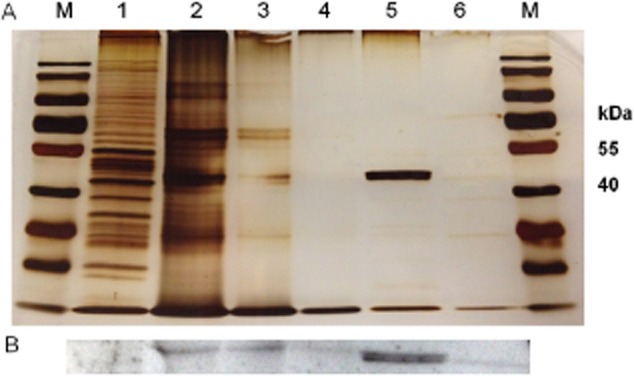
Detection of Ack (∼ 46 kDa): (A) SDS-PAGE (10%, silver staining) – 1 *C. glutamicum* 13AK; 2, undiluted reactor content; 3, 1:2 diluted reactor content; 4, 1:5 diluted reactor content; 5–6, *C. kluyveri* (exponential and stationary growth phase respectively); M, protein standard (PageRuler^TM^ prestained) (B) Western blot – 1, *C. glutamicum* 13AK*;* 2–4, reactor content; 5–6, *C. kluyveri*.

Furthermore, cell free extract of biogas reactor content (sample B, BR1) and corresponding controls were loaded on an SDS-PAGE (Fig. 1A) for subsequent Western blot analysis, which was conducted for the detection of Ack in the analysed biogas reactor content of BR1 (Fig. 1B). In all dilutions of cell free extracts from biogas reactor content (lanes 2–4), the same signal was detected as in cell free extracts of *C. kluyveri* DSM 555, which was used as positive control (lanes 5 and 6).

Quantification of the genes coding for Buk (*buk*) and But (*but*) was performed using genomic DNA from samples A–E from BR1 listed in Table 1. Neither *buk* nor *but* gene fragments were quantifiable in any sample by quantitative polymerase chain reaction (qPCR) assay because all used dilutions in the assay had the same crossing point (Cp) value (55.0 for *buk* and 42.6 for *but*). However, simultaneous quantification of the 16S rRNA used as reference gene was successful for all samples and every dilution used, in terms of sufficient calculation of standard curves.

Furthermore, possible transcription of the genes *buk* and *but* was checked using reverse transcriptase PCR (RT-PCR). No PCR product was amplified from respective cDNA for *buk* and *but* in the samples A and D, indicating that the transcription of both genes did not occur (Fig. S1).

**Figure 2:**
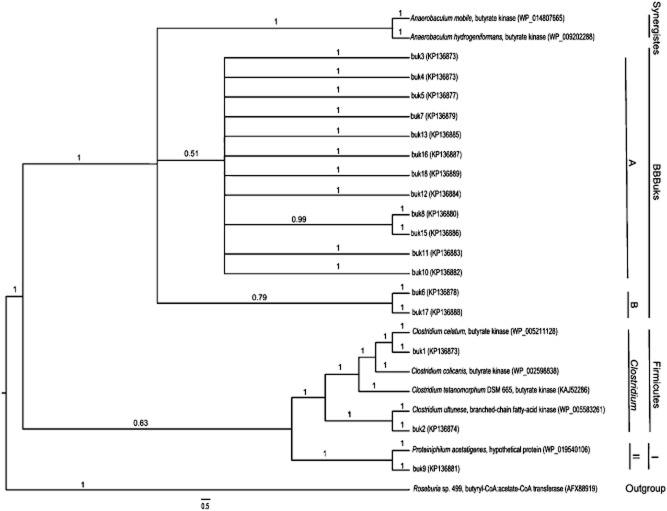
Phylogenetic tree based on amino acid sequences derived from *buk* nucleotide sequences. Sequences obtained and examined in this study are indicated with *buk*1-17. Used strains served as reference sequences. Accession numbers for the sequences used are given in brackets. I, Bacteroidetes II, Proteiniphilum. Bootstrap values (1 as 100%) are shown on branches. The scale bar represents the genetic distance (5 substitutions per 100 amino acids).

**Figure 3:**
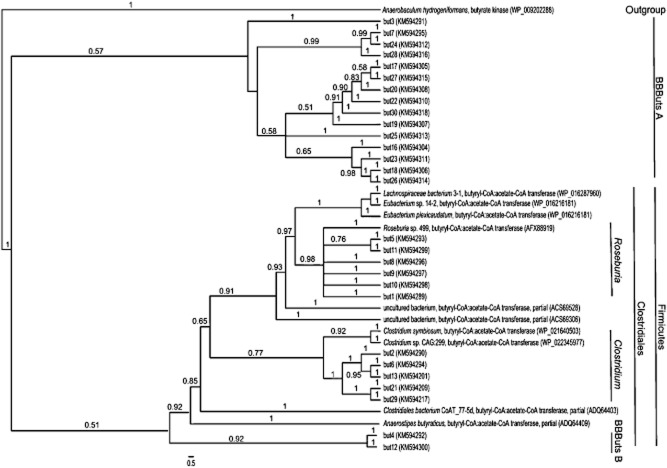
Phylogenetic tree based on amino acid sequences derived from *but* nucleotide sequences. Sequences obtained and examined in this study are indicated with *but* 1–28. Used strains served as reference sequences. Accession numbers for the sequences used are given in brackets. Bootstrap values (1 as 100%) are shown on branches. The scale bar represents the genetic distance (5 substitutions per 100 amino acids).

The results suggest that amplification of both conserved genes is possible, although the quantification was not successful. By using a standard cloning approach of *buk* and *but* gene fragments, 218 and 301 clones were obtained for *buk* and *but* respectively. The corresponding gene fragments were translated into respective amino acid sequences. Only 17 translated and meaningful *buk* sequences from initially 218 picked and analysed clones could be finally used for the phylogenetic tree (Fig. 2), leading to an efficiency of 7.8%. Three hundred one *E. coli* clones were analysed for the phylogenetic tree of *but* gene fragments (Fig. 3) with 144 potentially useful sequences, adding up to a theoretical efficiency of 47.8%. Amino acid sequences that did not group with any reference sequences were designated as butyrate-producing bacteria using Buk or But (BBBuks or BBButs) respectively. Considering the phylogenetic tree of *buk*, amino acid sequences of buk1 and buk2 cluster in the genus *Clostridium*. The amino acid sequences of Buk of *C. celatum* and a branched-chain fatty-acid kinase of *C. ultunese* have the highest sequence similarity to buk1 and buk2 respectively. The amino acid sequence of buk9 shows highest similarity to the reference sequence of a hypothetical protein of *Proteiniphilum acetatigenes*. BBBuks A and BBBuks B do not group in any reference phyla, indicating two new clades of butyrate-producing bacteria.

Thirteen of the 28 amino acid sequences derived from *but* fragments cluster in the phylum Firmicutes and particularly in the order Clostridiales. Furthermore, six and five of these sequences group in the genera *Roseburia* and *Clostridium* respectively. Hence, but4 and but12 cannot be assigned to any reference genus and form a new clade BBButs B. The remaining 15 sequences form apparently also a new clade BBButs A, not belonging to the phylum Firmicutes.

## Discussion

The aim of this study was to elucidate the activity and presence of acidogenic key enzymes Ack, Buk and But, and the corresponding genes *buk* and *but*, respectively, in the biogas process. Results of the specific enzyme activity assays of Ack, Buk and But indicate a stable substrate degradation within the sampled biogas reactor BR1, as there are hardly any fluctuations during the sampling period. Duncan and colleagues (2002) as well as Louis and colleagues (2004) also did report on specific enzyme activities of Ack, Buk and But using bacterial strains from the human large intestine. The determined specific enzyme activities were in a similar range as found in this study. The overall trend was that specific But activity was highest, followed by specific Ack activity, and specific Buk activity was lowest (Duncan *et al*., 2002; Louis *et al*., 2004). This tendency was also observed in the analysed samples of two further investigated biogas reactors (Table 2, Table S1). In an anaerobic environment, such as the swine intestinal tract, the enzymatic step for butyrate formation is commonly the activity of But rather than the activity of Buk (Levine *et al*., 2013) because bacterial strains tested positively for But activity were at the same time negative for Buk activity. This can also be supported by metagenomic data drawn from stool samples, in which the pathway of butyrate production by *but* as terminal gene was favoured over *buk* (Vital *et al*., 2014). Both findings match with the results obtained in this study. Latest results of Klang and colleagues (2015) showed that there was a correlation between the ammonium (NH_4_^+^-N) concentration and the diversity and composition of the microbial community. Similar findings were reported by Westerholm and colleagues (2011) to the effect that increasing ammonia concentration caused a shift in the acetogenic population in a mesophilic anaerobic digester. Ammonia concentration has a strong impact on the microbial community structure in anaerobic environments (Westerholm *et al*., 2011; Fotidis *et al*., 2013; Klang *et al*., 2015). Moreover, we show an influence of NH_4_^+^-N concentration on the enzymatic activities within different biogas reactors. Based on the collected data, we postulate that high NH_4_^+^-N and NH_3_ concentrations in biogas reactor content cause low enzyme activities of acidogenesis, and consequently a low amount of respective catalyst is present.

Western blot analysis was successfully used for the detection of enzymes in a biogas reactor, in this case Ack (Fig. 1B). So far, an unsuccessful attempt has been reported to detect enzymes (exoglucanase CelS from *C. thermocellum*) in samples from a biogas reactor (Köllmeier, 2013). Due to low Buk activity and lack of suitable antibody against But at the time of the study, Western blot analysis was neither performed using Buk nor But.

After determination of specific Buk and But activities in the sampled biogas reactor, the next step was the analysis of the quantity and the transcription of the corresponding gene fragments of Buk and But. Primer pairs targeting both mentioned gene fragments were introduced by Louis and colleagues (2004) and by Louis and Flint (2007) for butyrate-producing bacteria in the human colon. In this study, both qPCR and RT-PCR failed for the gene fragments *buk* as well as *but*. In the case of *buk*, the failed quantification on DNA level was not surprising due to the fact that specific Buk activity was barely measurable. In contrast, high specific But activity was found in all sampled biogas reactors. For this reason, quantification of *but* gene fragment was actually expected. Levine and colleagues (2013) also reported on butyrate-producing isolates from swine intestinal tract, which showed But enzyme activity. However, *but* genes could not be detected by using the same degenerate primer pair (BCoATscrF and BCoATscrR) used in this study. Furthermore, unsuccessful RT-PCR (Fig. S1) for both gene fragments *buk* and *but* indicates a restricted usage of the used primer pairs for genomic DNA or cDNA obtained from the sampled biogas reactor. Additionally, the used primer pairs need to be improved for this kind of samples and analyses. Anyhow, high Cp values in qPCR indicate an amplification of both gene fragments anyway, despite no possible quantification. Therefore, phylogenetic trees were constructed for either *buk* or *but* gene fragments to get an overview of butyrate-producing bacteria in the sampled biogas reactor (Figs 2 and 3). At the 16S rRNA and at the metagenome level, the presence of the phyla Firmicutes and Bacteroidetes was also previously reported for biogas reactors (Souidi *et al*., 2007; Schlüter *et al*., 2008; Liu *et al*., 2009; Kampmann *et al*., 2012; Bengelsdorf *et al*., 2013; Li *et al*., 2013; Stolze *et al*., 2015). In the reports at the 16S rRNA level, both phyla were the predominant bacteria in biogas reactors, with up to 58.9 and 35.4% of all analysed sequences respectively. In the anaerobic environment of the human intestinal tract, bacteria of the genera *Roseburia* and *Clostridium* were also found as butyrate producers at the *but* gene level and at the 16S rRNA level respectively (Duncan *et al*., 2002; Pryde *et al*., 2002; Louis *et al*., 2004; 2010; Louis and Flint, 2007; Hippe *et al*., 2010). In this study, four new clades of possible butyrate-producing bacteria were identified. So far, BBBuks A and B, and BBButs A seem to form each an unidentified clade of bacteria. Assigning these clades to any known phylum is challenging as most reports on microbial diversity in biogas reactors are based on 16S rRNA analyses rather than analyses with special biomarkers, such as the *buk* or the *but* gene. For example, members of the phylum Spirochaetes are in possession of a gene encoding Buk (Mavromatis *et al*., 2010) and were also found in low percentages in biogas reactors (Souidi *et al*., 2007; Liu *et al*., 2009; Stolze *et al*., 2015). In some cases, Proteobacteria were found in small numbers in biogas reactors (Fang *et al*., 2002; Souidi *et al*., 2007; Stolze *et al*., 2015) and in larger fractions in gastrointestinal tracts of different animals (Metzler-Zebeli *et al*., 2010; Wüst *et al*., 2011; Van den Abbeele *et al*., 2013). Furthermore, some Proteobacteria, such as *Burkholderia vietnamiensis* G4 (NCBI Gene ID: 4953143), *Shewanella* sp. MR-7 (NCBI Gene ID: 4257334) or *Sphingomonas wittichii* RW1 (NCBI Gene ID: 5198581), are actually carrying a gene encoding But. Apparently, BBButs B clustered in the phylum Firmicutes (Fig. 3). For example, the genera *Faecalibacterium* or *Coprococcus* were found in the human colon as butyrate producers using But (Duncan *et al*., 2002; Pryde *et al*., 2002; Hippe *et al*., 2010; Louis *et al*., 2010). Quantification or further analysis of genes encoding Ack was not conducted due to so far missing conservative primers.

Despite unsteady substrate supply of BR1 (Table 1), stable and high specific activities of Ack and But were found, indicating high and stable substrate degradation rates and respective conversion rates of organic acids to methane. Concomitant low concentrations of acetate and butyrate (Table 1) also point to no high acid accumulation in the sampled biogas reactor. Furthermore, high ammonia and ammonium concentrations have a strong impact on specific enzymatic activities. The higher these concentrations, the lower are the specific enzymatic activities of acidogenesis. Inhibition of the acidogenesis, which provides the substrates for the methanogens, may also impact the methane formation in biogas reactors. Additionally, no quantification of *buk* gene fragments, low efficiency regarding the phylogenetic tree of *buk* amino acid sequences and low Buk activity support the assumption of butyrate production in the sampled biogas reactor almost exclusively by the activity of But.

## Experimental procedures

### Biogas plant and sampling

A stably performing mesophilic full-scale biogas reactor fed with maize silage, cattle manure and poultry dry manure located near Bonn (Troisdorf, Germany) was chosen for protein and genome studies (biogas reactor 1, BR 1). The organic loading rate of the reactor was on average 4.8 kg VS m^−3^ d^−1^ (VS, volatile solids) with a total solid feeding of 14 800 kg per day and a hydraulic retention time of 85 days. The methane content accounted to 53% (v/v) of the 330–350 m^3^ produced biogas per hour. Additionally, two further mesophilic full-scale similarly fed biogas reactors were sampled for enzyme activity studies (BR 2 and BR 3). The organic loading rates of BR 2 and BR 3 were on average 3.87 and 7.17 kg VS m^−3^ d^−1^, with hydraulic retention times of 126 and 63 days respectively. In both reactors, the methane content was comparable to BR 1. For each sample, 1 l reactor content was drawn at different time points during the study and transported in cooled boxes. All samples are listed in Table 1, as well as the operating conditions of the biogas plants. Samples were provided by Bioreact GmbH (Troisdorf, Germany), and standard analyses were performed by an accredited laboratory (Bonalytik GmbH). For enzyme studies, 50 ml reactor content were centrifuged (4000 × *g*, 15 min, 4°C), washed twice with 25 ml PBS buffer (1.5 mM KH_2_PO_4_, 4.2 mM Na_2_HPO_4_, 137 mM NaCl, 1.5 mM KCl) and stored at −20°C. For DNA and RNA studies, 25 ml ethanol (96%, v/v) was added to 25 ml reactor content and stored at −20°C.

### Extraction of total protein from biogas reactor content

For the aerobic determination of specific enzyme activities of Ack, Buk and But, as well as Western blot analysis of Ack, samples stored at −20°C were thawed on ice by the addition of one volume of corresponding enzyme assay reaction buffer (see below). Homogenization of the cells was carried out by using an ultrasonic pulser (Sonifier 250, Branson, Dietzenbach, Germany) with the following adjustments: 30 s pulsing at 60–70% intensity, 30 s break, 15 cycles (modified; Refai *et al*., 2014). Cell debris was pelleted by centrifugation (4000 × *g*, 20 min, 4°C). The supernatant was filtrated using folded filters (particle retention 7 μm, 595.5 grade) and centrifuged (11 000 × *g*, 5 min, 4°C). The obtained cell free extract was used for enzyme assays and Western blot analysis.

*Corynebacterium glutamicum* ATCC 13032 13AK with defective Ack activity (Wendisch *et al*., 1997) and *Clostridium kluyveri* DSM 555 (DSMZ, Braunschweig, Germany) were used as negative and positive controls in the enzyme studies respectively. Both strains were cultivated to mid-exponential or stationary growth phase aerobically in CGXII minimal medium (Keilhauer *et al*., 1993) with 1% glucose or anaerobically in medium 52 (DSMZ) respectively. Harvested cells were washed twice with corresponding enzyme assay reaction buffer and stored at −20°C. Mechanical cracking of the cells was achieved by using a homogenisator (three cycles, 45 s, 6500 m s^−1^), followed by centrifugation (11 000 × *g*, 30 min, 4°C) to obtain cell free extracts for further use.

### Determination of protein concentrations and enzyme activities

Protein concentrations of cell free extracts were determined by using the BCA Protein Assay Kit (Thermo Fisher Scientific, Rockford IL, USA) following the manufacturer’s instructions.

Samples A-G of BR 1 were used for the determination of the enzyme activities of Ack and Buk, and samples B-G of BR 1 were used for the determination of the enzyme activity of But. Both samples of BR 2 and BR 3 were used for all three enzymatic assays. In all cases, at least two dilutions of each sample were measured twice to assure linearity of enzyme activity. Enzyme assays were performed routinely in a cuvette at 30°C. Ack and Buk activity assays (Nakajima *et al*., 1978) comprised reaction buffer (100 mM Tris, pH 7.2, 5 mM MgCl_2_), 0.5 mM NADP^+^, 3.0 mM ADP, 2.0 mM glucose, and 10 μl hexokinase/glucose-6-phosphate dehydrogenase (3 mg ml^−1^; Roche Diagnostics, Filderstadt, Germany). In a total volume of 1.0 ml, the reaction was initiated by the addition of 5 mM acetyl- or butyryl-phosphate for the estimation of specific Ack or Buk activity respectively. The increase of absorbance of NADPH was followed at 340 nm with an extinction coefficient ε = 0.063 mM^−1^ cm^−1^. But activity assay (Buckel *et al*., 1981) comprised reaction buffer (100 mM potassium phosphate, pH 7.0), 200 mM sodium acetate, pH 7.0, 1.0 mM oxaloacetate, 1.0 mM DTNB, and 10 U citrate synthase (Sigma-Aldrich, Steinheim, Germany). In a total volume of 1.0 ml, the reaction was initiated by the addition of 1.0 mM butyryl-CoA. The increase of absorbance of TNB^−^ was followed at 412 nm with an extinction coefficient ε = 14.2 mM^−1^ cm^−1^.

### Western blot

For Western blot analysis, equal amounts (200 μg) of cell free extracts (sample B of BR1, controls) and respective dilutions (1:2 and 1:5) were loaded onto an SDS-PAGE (10%, v/v) to detect Ack in biogas reactor content. Protein was silver-stained or transferred onto nitrocellulose membranes by electro blotting and blocked with 2% BSA over night. Blots were incubated with primary antibody (Ack antibody; Biorbyt, Cambridge, UK) and with secondary antibody [polyclonal goat anti-rabbit (HRP, horse radish peroxidase) antibody; Dakocytomation, Glostrup, Denmark], each for 1 h. Signals were detected by enhanced chemoluminescence (Pierce ECL Western Blotting, Thermo Fisher Scientific).

### Extraction of total DNA and RNA from biogas fermenter content

Analysis of the functional genes *buk* (encoding Buk), *but* (encoding But) and 16S rRNA as reference gene was achieved by qPCR, RT-PCR and PCR. Nucleic acids were obtained by using extraction kits for DNA (NucleoSpin® Soil, Macherey-Nagel GmbH & Co. KG, Düren, Germany) from samples A–E of BR1 (Table 1) and for RNA (ZR Soil/Fecal RNA MicroPrep™, Zymo Research Corp., Irvine CA, USA) from samples A and D of BR1, respectively, by following the manufacturer’s instructions. Additionally, extracted total RNA was treated with DNase I (Invitrogen GmbH, Karlsruhe, Germany).

### qPCR, RT-PCR, PCR

Primer sets used in this study for the analysis of the functional genes *buk*, *but* and 16S rRNA were applied for either qPCR, RT-PCR or PCR: Buk gene (*buk* fragment, app. 740 bp: BUKrev1, CAAGCTCRTCNACNACNACNGGRTCNAC, PTBfor2 CAAATTAAAGGNTGYRTNRTNGAYGGNCC; Louis *et al*., 2004), But gene (*but* fragment, app. 540 bp: BCoATscrF GCNGANCATTTCACNTGGAAYWSNTGGCAYATG, BCoATscrR CCTGCCTTTGCAATRTCNACRAANGC; Louis and Flint, 2007), and 16S rRNA gene (16S rRNA fragment, app. 450 bp: U515-532 GTGYCAGCMGCCGCGGTA, E969-984 GTAAGGTTCYTCGCGT; Wang and Qian, 2009). Quantification of *buk*, *but* and 16S rRNA gene fragments was performed in the LightCycler® 480 (Roche Diagnostics) using KAPA™ SYBR® FAST Master Mix (Peqlab Biotechnologie GmbH, Erlangen, Germany) with 1 cycle of 95°C for 3 min, 60 cycles of 95°C for 10 s, 60°C for 20 s and 72°C for 10 s. Standard curves derived from applying different dilutions of total DNA. All data were evaluated with LightCycler® 480 software.

The amplification cycle performed for *buk* and *but* fragments was 1 cycle of 95°C for 5 min, 40 cycles of 95°C, 55°C, and 72°C for 1 min each, and 1 cycle of 72°C for 10 min. Total DNA (500 ng μl^−1^) or cDNA served as templates, and ReproFast polymerase (Genaxxon Bioscience, Ulm, Germany) was used.

RT-PCR was conducted with random primers, SuperScript™ III Reverse Transcriptase and First Strand Synthesis Buffer (5×) (Invitrogen GmbH) on DNA-free total RNA.

### Clone library construction and sequence analysis

In order to get an overview of butyrate-producing bacteria in biogas reactors, two possible metabolic pathways, with Buk or But as key enzymes, were investigated by quantification and phylogenetic analysis. The PCR fragments of *buk* and *but* genes were purified and cloned into pDrive cloning vector (Qiagen, Venlo, Netherlands) and transformed into *Escherichia coli* XL1-Blue MRF’ competent cells (Stratagene, La Jolla, CA, USA) for blue-white screening. One hundred six clones for *buk* fragment and 224 clones for *but* fragment were sequenced with standard M13 primer set (M13F GTAAAACGACGGCCAGT, M13R AACAGCTATGACCATG) by GATC Biotech AG (Konstanz, Germany). After elimination of chimeric sequences or sequences with low quality, 17 and 144 sequences were used for further applications respectively. Seventeen nucleotide sequences of *buk* fragment were translated into amino acid sequences using ExPASy translate tool (SIB Bioinformatics Resource Portal). One hundred forty-four obtained *but* fragment sequences were clustered using Clone Manager Suite leading to 10 clusters. Up to three randomly chosen sequences from each cluster were translated into amino acid sequences also using ExPASy translate tool, yielding 28 sequences. For each deduced data set of *buk* and *but* fragment sequences, multiple sequence alignments (MSAs) were calculated using mafft (Katoh and Standley, 2013). The resulting MSAs were checked visually. The reconstruction of phylogenetic trees based on each of these MSAs was performed with the programme MrBayes version 3.2.3 (Ronquist *et al*., 2012). The default settings recommended in the respective manual for tree reconstruction were used for the analyses. All used sequences for phylogenetic trees were submitted to GenBank under accession numbers KP136873 to KP136889 and KM594289 to KM594320 respectively.

## Supporting Information

**Fig. S1.** Detection of *buk* (A, 740 bp), *but* (B, 540 bp) and 16S rRNA gene (C, 450 bp) via RT-PCR: agarose gel (2%) of PCR products – Lane: M, 50 bp DNA ladder (GeneRuler^TM^); 1 and 3, cDNA; 2 and 4, RNA; 5, positive control (DNA); 6, negative control (H_2_O).

**Table S1.** Specific enzyme activities in U mg^−1^ protein, enzymatic volume activity in mU g^−1^ oDS of Ack, Buk and But in cell free extracts of biogas reactor content BR 1, BR 2 and BR 3. Values of ammonia concentrations for NH_4_^+^-N and NH_3_ in g l^−1^.
