# Supplementary figures and images for: Analysis of the key enzymes of butyric and acetic acid fermentation in biogas reactors
Source: Microb Biotechnol. 2015 Jun 18;8(5):865–73. doi: 10.1111/1751-7915.12299 (PMC4554474; doi:10.1111/1751-7915.12299)

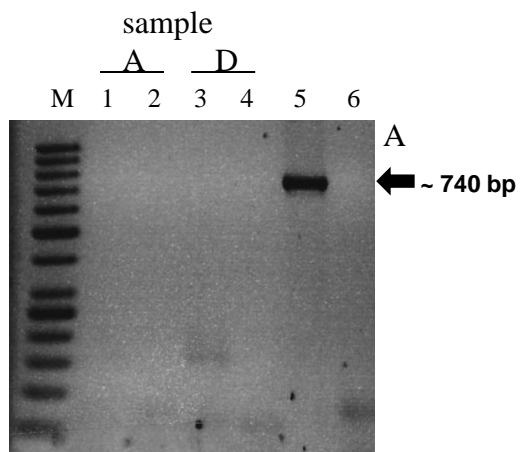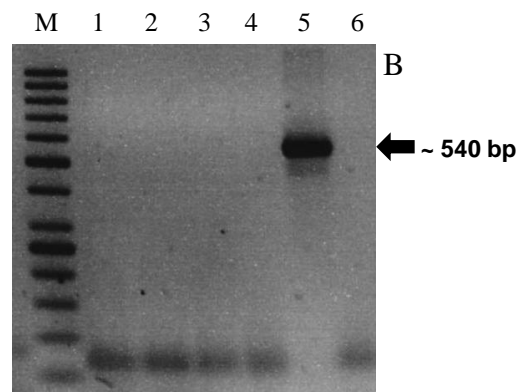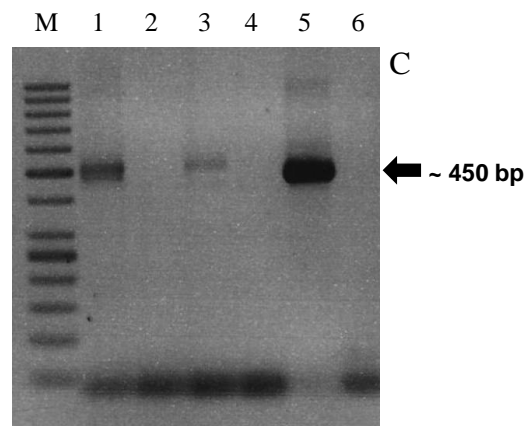

Supplement: Supplementary file 1 [file mbt20008-0865-sd1.pdf]
