# Supplementary material for: Analysis of the key enzymes of butyric and acetic acid fermentation in biogas reactors
Source: Microb Biotechnol. 2015 Jun 18;8(5):865–73. doi: 10.1111/1751-7915.12299 (PMC4554474; doi:10.1111/1751-7915.12299)
Supplement: Supplementary file 2 [file mbt20008-0865-sd2.docx]

Table S1: Specific enzyme activities in U mg^-1^ protein, enzymatic volume activity in mU g^-1^ oDS of Ack, Buk, and But in cell free extracts of biogas reactor content BR 1, BR 2, and BR 3. Values of ammonia concentrations for NH_4_^+^-N and NH_3_  in g L^-1^.

|  | **U mg^-1^ protein** | | | **mU g^-1^ oDS** | | | **NH_3_ [g L^-1^]^3^** | **NH_4_^+^-N [g L^-1^]** |
| --- | --- | --- | --- | --- | --- | --- | --- | --- |
| sample | Ack | Buk | But | Ack | Buk | But |  |  |
| A | 0.50 | 0.01 | n.d.^1^ | 30.79 | 5.05 | n.d.^1^ | 0.33 | 3.14 |
| B | 0.48 | 0.01 | 6.34 | 35.28 | 0.79 | 2429.92 | 0.51 | 4.38 |
| C | 0.46 | 0.01 | 6.66 | 92.85 | 2.23 | 1675.15 | 0.77 | 4.48 |
| D | 0.41 | 0.01 | 7.61 | 407.08 | 11.56 | 5391.84 | 0.76 | 4.90 |
| E | 0.29 | 0.01 | 6.94 | 119.77 | 2.86 | 4681.36 | 0.43 | 3.37 |
| F | 0.24 | 0.01 | 4.53 | 145.29 | 4.29 | 4406.81 | 0.43 | 3.20 |
| G | 0.47 | 0.01 | 3.46 | 437.63 | 8.67 | 4536.14 | 0.34 | 3.05 |
| **mean** | **0.41** | **0.01** | **5.92** | **181.24** | **5.06** | **3853.54** | **0.51** | **3.79** |
| **s.d.^2^** | **0.10** | **0.00** | **1.59** | **170.08** | **3.81** | **1455.73** | **0.18** | **0.77** |
| A1 | 0.14 | 0.007 | 3.18 | 117.88 | 6.03 | 3641.86 | 0.67 | 4.28 |
| A2 | 0.33 | 0.007 | 3.29 | 286.50 | 6.31 | 3534.49 | 0.76 | 4.54 |
| **mean** | **0.23** | **0.007** | **3.24** | **202.19** | **6.17** | **3588.18** | **0.71** | **4.41** |
| B1 | 0.62 | 0.017 | 6.91 | 277.13 | 7.57 | 4304.31 | 0.04 | 0.92 |
| B2 | 1.35 | 0.037 | 8.37 | 329.30 | 9.03 | 2306.50 | 0.06 | 1.49 |
| **mean** | **0.99** | **0.027** | **7.64** | **303.22** | **8.30** | **3305.41** | **0.05** | **1.21** |

^1^ not determined; ^2^ standard deviation; ^3^ calculated (Angelidaki and Ahring, 1993)
